# Japan useful medication program for schizophrenia (JUMPs)-long-term study on discontinuation rate, resolution and remission, and improvement in social functioning rate associated with atypical antipsychotic medications in patients with schizophrenia

**DOI:** 10.1186/1471-244X-13-243

**Published:** 2013-10-03

**Authors:** Jun Ishigooka, Kazuyuki Nakagome, Tetsuro Ohmori, Nakao Iwata

**Affiliations:** 1 Department of Psychiatry, Tokyo Women’s Medical University, School of Medicine, Shinjuku-ku, Tokyo, Japan; 2 Translational Medical Center, National Center of Neurology and Psychiatry, Kodaira, Tokyo, Japan; 3 Department of Psychiatry, The University of Tokushima Graduate School, Tokushima, Japan; 4 Department of Psychiatry, Fujita Health University School of Medicine, Toyoake, Aichi, Japan

**Keywords:** Schizophrenia, Effectiveness study, Aripiprazole, Blonanserin, Paliperidone

## Abstract

**Background:**

It is desirable to establish evidence for the selection of antipsychotics from the viewpoint of recovery of social activity in individual patient with schizophrenia receiving medication. From this perspective, awareness of the importance of studies about drug effectiveness on treatment discontinuation rate, remission rate, and improvement in QOL has grown recently. In Western countries, numerous reports are available in effectiveness studies, which are related to olanzapine and risperidone primarily, whereas evidence for other second-generation antipsychotics (SGAs) is poor. In Japan, no effectiveness study has been reported: thus, it is desirable to collect data that will serve as evidence for selection of the 3 SGAs approved after olanzapine.

**Methods:**

The present study was a long-term effectiveness study under healthcare setting in Japan. It was designed as an open-label, multicenter, randomized, comparative study involving 104-week oral treatment with 1 of the 3 drugs (aripiprazole, blonanserin, and paliperidone) in patients with schizophrenia aged 20 years or over who required antipsychotic medication or switching of the current medication to others for reasons such as lack of efficacy and intolerability. The primary endpoint is treatment discontinuation rate for any causes. The secondary endpoints include remission rate, improvement of social activity, alleviation, aggravation or recurrence of psychiatric symptoms, and safety. The target number of subjects was set at 300.

**Discussion:**

Because this study is expected to yield evidence regarding the selection of antipsychotics for facilitating the recovery of social activity in patients with schizophrenia, it is considered highly valuable to perform this effectiveness study under ordinary healthcare setting in Japan.

**Trial registration:**

UMIN Clinical Trials Registry 000007942

## Background

Clinical studies performed during the development of new antipsychotics (efficacy studies) often involve many eligibility criteria for the selection of candidates and tend to be confined to small patient groups. Furthermore, in efficacy studies, the magnitude of changes in psychiatric symptom score during short periods of time and adverse reactions are evaluated independently. Because of these features of study design, it is difficult to generalize the results of efficacy studies readily into rationales for the selection of antipsychotics for schizophrenia in routine clinical practice [1]. In contrast, the goals of long-term treatment for schizophrenia encompass improvement in the social activity and in the Quality of Life (QOL) of patients. In this sense, effectiveness studies that can yield new findings regarding the long-term outcome of treatments have become increasingly important. In Western countries, evidence partially supporting the selection of antipsychotics has been accumulating from studies that compared the long-term outcome primarily of olanzapine and risperidone, as well as other second-generation antipsychotics (SGAs) and first-generation antipsychotics (FGAs) [2,3]. In Japan, however, no large-scale effectiveness study has been carried out. Moreover, sufficient data on the long-term outcome of treatment with aripiprazole, blonanserin, and paliperidone, that were marketed relatively recently, are unavailable in Japan and in Western countries. Thus, it is desirable to obtain evidence for the selection of these 3 drugs in Japan.

Under such circumstances, we planned a long-term (2-year) dosing study to evaluate the effectiveness of aripiprazole, blonanserin, and paliperidone on discontinuation rate, remission rate and improvement of social activity in patients treated with these drugs (Japan Useful Medication Program for schizophrenia: JUMPs).

## Methods

### Setting

**Figure 1:**
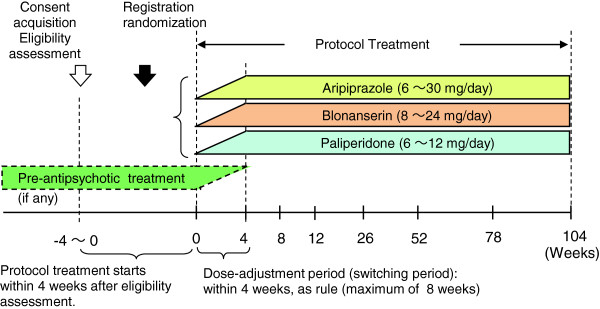
**Study outline (no legend in Figure**1).

This is an open-label, multicenter, randomized study involving parallel-group comparison of the treatment discontinuation rate, remission rate, and improvement of social activity during 2-year treatment with aripiprazole, blonanserin, or paliperidone in patients with schizophrenia (Figure 1). This study complies with the Declaration of Helsinki and Good Clinical Practice (GCP). The study starts after having the approval for the study granted by each institutional review board at participating clinical site. If the participating sites have not established own review board, the site requested the review for the study from the review board at other medical institute and obtained the approval (the Ethics committee of Tokyo Women’s Medical University Hospital, the Ethics Committee of the National Center of Neurology and Psychiatry, the Ethics Committee of Fujita Health University Hospital, the Ethics Committee of Tokushima University Hospital, Shinagawa Clinic Institutional Review Board).

### Subjects

Patients eligible for the study are outpatients with schizophrenia aged 20 years or over who were diagnosed according to Diagnostic and Statistical Manual of Mental Disorders, 4th edition text revision (DSM-IV-TR), those requiring new treatment with antipsychotics or requiring switching of the current antipsychotics to others for reasons such as lack of efficacy and intolerability. Details of the inclusion and exclusion criteria are shown in Table 1. Prior to participating in the study, written consent is obtained from each patient.

**Table 1:** Patient inclusion and exclusion criteria

**Criteria**	
Patients satisfying all of the following requirements are eligible.	
•	Outpatients aged 20 years or over at the time of consent acquisition
•	Patients with schizophrenia satisfying the DSM-IV-TR diagnostic criteria
•	Patients requiring antipsychotic treatment or switching from the current antipsychotic medication to others because of lack of efficacy, intolerability, or other reasons
•	Patients capable of issuing consent to participate in the study in writing
**Exclusion criteria**	
Patients falling under any of the following criteria are excluded from this study.	
•	Patients having a history of allergy to any component of the test drugs or risperidone
•	Patients under strong influence from drugs that suppress the central nervous system, such as barbiturate derivatives
•	Patients receiving adrenaline
•	Patients receiving azole antifungal agents (excluding topical preparations) or HIV protease inhibitors
•	Patients with moderately or severely compromised renal function (creatinine clearance less than 50 mL/min at the time of eligibility assessment)
•	Patients with poorly controlled diabetes mellitus (HbA1c (NGSP) over 8.4% at the time of eligibility assessment)
•	Patients presenting with severe symptoms despite sufficient antipsychotic treatment or patients having a history of clozapine therapy
•	Patients having other psychiatric disease in addition to schizophrenia
•	Patients with complication of Parkinson’s disease
•	Patients presenting with malignant syndrome or similar symptoms or patients with water poisoning
•	Patients having signs of physical exhaustion, such as dehydration and malnutrition
•	Patients having malignant tumor requiring treatment
•	Patients with a history of alcohol or drug abuse
•	Pregnant or possibly pregnant women, lactating women, or women desiring to become pregnant during the study period
•	Patients likely to attempt suicide (CGI-SS class 4 or higher (severe tendency for suicide))
•	Other patients judged by the physician in charge as inappropriate for the study

*DSM*-*IV*-*TR*, Diagnostic and Statistical Manual of Mental Disorders, 4th edition, text revision; *HIV*, human immunodeficiency virus; *HbA*1*c*, glycohemoglobin *A*1*c*; *NGSP*, National Glycohemoglobin Standardization Program; *CGI*-*S*S, Clinical Global Impression-Suicide Scale.

The target number of subjects was set at 300, because the major objective of this study is to explore the discontinuation rate associated with SGA treatment.

### Design

Patients having issued consent to the study and eligible for participating in the study are randomly allocated to 1 of the 3 groups treated with aripiprazole, blonanserin, or paliperidone by the central registration center, to start the protocol treatment (Figure 1). Each drug is administered orally at an initial dose level or below the dose level approved for each drug (aripiprazole, <12 mg/day; blonanserin, <8 mg/day; and paliperidone, <6 mg/day). For subjects having received antipsychotics before the start of the protocol treatment, the preceding antipsychotics are switched to the protocol treatment during the dose adjustment period for 4 weeks, as a rule (8 weeks at maximum). During the study period except for the dose adjustment period, the dosage of each study drug may be adjusted within the dosage approved (aripiprazole, 12–30 mg/day; blonanserin, 8–24 mg/day; and paliperidone, 6–12 mg/day), depending on the condition of individual patient. Concomitant use of any antipsychotics other than the study drugs is prohibited (in case of need, concomitant use of 1 oral-dose drug is permitted at dose levels within the approved range, up to 4 doses in 4 weeks). Anti-anxiety drugs or antidepressants used concomitantly before the start of the protocol treatment are continued during the study period without changing the drugs or their dosage. Adding these drugs during the study period newly is not allowed. However, in cases needed, it is permitted to use lorazepam concomitantly during the study period at a frequency up to 10 doses in 4 weeks and at a daily dose level within the approved range (3 mg/day at maximum). The study drug-treatment period will be 104 weeks. No restriction is placed on treatment after discontinuation of the protocol treatment. However, it is recommended to select 1 of the 2 study drugs left which was not used in the protocol treatment.

Table 2 lists the parameters and timing of observations and tests. Observations and tests are conducted before and 8, 12, 26, 52, 78, and 104 weeks after the start of the protocol treatment (for subjects already having received antipsychotic treatment before the start of the protocol treatment, the tests and observations will also be performed upon completion of the switching from the preceding treatment to the protocol treatment). The comprehensive capacity to give informed consent of each patient is evaluated quantitatively using the Japanese version of the MacArthur Competence Assessment Tool for Clinical Research (MacCAT-CR) [4].

**Table 2:** Observations and tests

	**Eligibility assessment**	**Protocol treatment**							
Week	-4 to 0	0	^*^	8	12	26	52	78	104
Doctor’s check	○	○	○	○	○	○	○	○	○
Height^†^, body weight	○					○	○	○	○
Blood test^‡^	○	○		○	○		○		○
PANSS^§^		○	○	○	○	○	○	○	○
CGI-SS	○		○	○	○	○	○	○	○
CGI-S		○	○						
CGI-I				○	○	○	○	○	○
MacCAT-CR^||^ (if possible)		○							
Social activity (PSP, EQ-5D)		○	○			○	○		○
DIEPSS		○	○	○	○	○	○	○	○
Adverse events			○	○	○	○	○	○	○

*PANSS*, Positive and Negative Syndrome Scale; *CGI*-*SS*, Clinical Global Impression-Suicide Scale; *CGI*-*S*, Clinical Global Impression-Severity; *CGI*-*I*, Clinical Global Impression-Improvement; Mac*CAT*-*CR*, MacArthur Competence Assessment Tool for Clinical Research; *PSP*, Personal and Social Performance Scale; *EQ*-5*D*, Euro Quality of life-5 dimension; *DIEPSS*, Drug-Induced Extrapyramidal Symptoms Scale.

In case of study discontinuation, all observations and tests except for MacCAT-CR will be performed.

^*^ At the time of start of single-drug therapy.

^†^ Measured only at the time of eligibility assessment.

^‡^*Cr* (serum creatinine), *HbA*1c (glycohemoglobin *A1c),WBC*, *RBC*, hemoglobin, hematocrit, platelet count, *AST* (aspartate aminotransferase), *ALT* (alanine aminotransferase), *CK* (creatine kinase), blood glucose, prolactin.

^§^ Remission is evaluated in accordance with the Andreasen definition (rating below mild on all 8 items of the PANSS for 6 consecutive months or more).

^||^ Assessed at the latest by Week 12.

### Outcome measurements

The primary endpoint of the study is the discontinuation rate of the protocol treatment for any causes including lack of efficacy and intolerability. Secondary endpoints are as follows: (1) remission rate according to the definition of Andreasen (rating below mild on all 8 items of the Positive and Negative Syndrome Scale (PANSS) for 6 consecutive weeks or more) [5], (2) social activity score (Personal and Social Performance Scale (PSP), Euro QOL-5 dimension (EQ-5D)), (3) alleviation of psychiatric symptoms in terms of PANSS score, (4) psychiatric symptom aggravation and recurrence rate based on PANSS, Clinical Global Impression-Improvement (CGI-I), and Clinical Global Impression-Suicide Scale (CGI-SS) scores (Table 3), (5) incidence of adverse events, and (6) Drug-Induced Extrapyramidal Symptoms Scale (DIEPSS) score [6]. In addition, the percentage of patients in whom multiple antipsychotic medication is switched to monotherapy, and the influence of the comprehensive capacity of patients on improvement of social activity, will also be investigated.

**Table 3:** **Criteria for the evaluation of psychiatric symptom aggravation**/**recurrence**

**Satisfaction of at least 1 of the following 4 requirements**	
1)	CGI-I is 5 (minimally worse) or higher, and at least 1 of A and B is satisfied
	A. Scored 5 (moderate severe) or higher in any of the following PANSS items, i.e., “disturbed concept integration (P2)”, “hallucination-based behavior (P3)”, “doubt/persecutory sense (P6)”, and “unnatural thought content (G9)” with a score on such item(s) being higher by 2 or more points from the baseline score
	B. Scored 5 (moderate severe) or higher in any of the following PANSS items, i.e., “disturbed concept integration (P2)”, “hallucination-based behavior (P3)”, “doubt/persecutory sense (P6)”, and “unnatural thought content (G9)”, with a total score on these 4 items being higher by 4 or more points from the baseline score
2)	Hospitalized based on psychiatric symptom aggravation
3)	CGI-SS Part 1 rating of 4 (severely suicidal) or 5 (attempted suicide), or CGI-SS Part 2 rating of 6 (much worse) or 7 (very much worse)
4)	Behaved violently, causing clinically serious self-injury, injury to other people, or destruction of objects

### Statistical analyses

For the primary endpoint analysis, we estimate time to discontinuation of the protocol treatment using the Kaplan-Meier method. The percentage of corresponding cases at each point through the study period is calculated regarding the remission rate, aggravation and recurrence rate based on psychiatric symptoms, CGI-I score, and incidence of adverse events. The change from the point before starting the protocol treatment to each point is calculated regarding PANSS, social activity evaluation (PSP, EQ-5D), CGI-SS, and DIEPSS scores. The level of significance is set at 5% with two-tailed. The confidence interval is estimated with 95%.

## Discussion

Efficacy studies on antipsychotics can be characterized by the enrollment of small patient groups, short duration, separate evaluation of the magnitude of change in psychiatric symptom scores and adverse reactions. The results of efficacy studies exhibiting these characteristics are not always consistent with the usefulness of antipsychotics experienced by physicians in clinical practice. For this reason, there is a growing awareness of the importance of the long-term assessment using effectiveness studies: to this effect, numerous large-scale studies, such as the Clinical Antipsychotic Trials of Intervention Effectiveness (CATIE), have been carried out in Western countries [2,3,7-9]. In these studies, comparisons were made among SGAs or between SGAs and FGAs, yielding various findings that are useful for the selection of drugs (primarily risperidone, olanzapine, and quetiapine) based on long-term outcome. In the case of drugs such as aripiprazole, blonanserin, and paliperidone, which were marketed relatively recently in Japan, sufficient long-term outcome data have not been collected, even in Western countries.

Aripiprazole exhibits not only serotonin 5-HT2A receptor blocking activity, but also dopamine D2 and serotonin 5-HT1A receptor partial agonist activity, which is an activity that any other antipsychotics do not possess. Aripiprazole has a low adrenaline α1 and histamine H1 receptor blocking activity and no muscarinic M1 receptor blocking activity. It is less likely to exert sedative effects, to cause weight gain, or to affect the metabolic system [10,11]. Blonanserin has dopamine D2 and serotonin 5-HT2A receptor blocking activity but, unlike other SGAs, has higher affinity for dopamine D2 receptor than for serotonin 5-HT2A receptor. Therefore, it exhibits characteristics of dopamine-serotonin antagonists. Much is expected of this drug regarding its effects on acute-stage symptoms. Its affinity for adrenaline α1, serotonin 5-HT2C, histamine H1, and muscarinic M1 receptors is low, suggesting a lower likelihood of sedative action, weight gain, and influence on the metabolic system [12]. Paliperidone is a major active metabolite of risperidone that has D2 receptor blocking activity and a more potent 5-HT2A receptor blocking activity and serves as a serotonin-dopamine antagonist [13]. The preparation of oral paliperidone adopts a drug-delivery control system that uses osmotic pressure, enabling the administration of treatment once a day [14]. Similar to risperidone, paliperidone is expected to be particularly effective against hallucination and delusion at the acute stage of schizophrenia, however, this drug is relatively likely to cause extrapyramidal symptoms and elevation in blood prolactin levels [15]. Unlike the other SGAs, paliperidone is almost not affected by the enzymes involved in hepatic metabolism and is excreted primarily (60%) in the unchanged form into the urine, allowing the use of this drug in patients with mildly or moderately compromised hepatic function [16]. Thus, the 3 study drugs differ in pharmacological profiles related to receptor affinity and have varying metabolic and formulation characteristics. In previous placebo-controlled and active-drug-controlled short-term comparative studies that used changes in psychiatric symptom scores as indicators, these drugs exhibited an efficacy that was comparable to that of the other SGAs and were found to have no noteworthy safety-profile problems compared with the other SGAs [17-22]. Various disabilities arise from schizophrenia that exhibit largely unmet needs. Therefore, we may say that the collection of domestic evidence on these 3 drugs, which are marketed in Japan, is quite valuable.

The present work was designed as a study aimed at collecting evidence for the selection of drugs used for the treatment in patients with schizophrenia in clinical practice. The inclusion criteria were set so that the study can enroll not only patients having responded poorly to previous antipsychotic treatment, but also patients with poor tolerance to the antipsychotics used currently, and patients having received no antipsychotics. The evaluation of long-term outcome will use treatment discontinuation rate and remission rate as indicators for the following reasons: (1) this kind of evaluation emphasizes effectiveness and encompasses elements such as symptom control, safety, social activity improvement, and QOL improvement, thus requiring realistic, simple and quantitative indicators; and (2) recent naturalistic studies revealed the necessity of ensuring adherence to the dosing instructions as an indispensable factor to obtain effectiveness above a certain level, and the necessity of estimating the extent of social activity recovery, which is the final goal of schizophrenia treatment [23,24]. The recurrence of schizophrenia can reduce the social activity of patients. Therefore, it is important to determine how to prevent its recurrence. In addition, in the present study, the treatment discontinuation rate as a marker of adherence was adopted as the primary endpoint. The evaluation of this parameter is simplified by including discontinuation of treatment for any reason. The results of such analyses are expected to serve as the evidence for the selection of antipsychotics to be used under diverse healthcare setting in Japan. The analysis using the remission rate as an indicator of the potential for social activity recovery in patients with schizophrenia is meaningful. To evaluate the remission rate, we adopted the Andreasen definition (rating below mild on all 8 items of the PANSS for 6 consecutive months or more) [5]. There are no uniform criteria for judging the recurrence of schizophrenia. However, hospitalization based on symptom aggravation or changes in PANSS or CGI scores is often used for the evaluation of relapse [25]. Consequently, in the present study, these indicators are used in the evaluation of the recurrence. For the evaluation of social activity improvement, assessments of QOL using PSP and EQ-5D will also be carried out. In contrast with the previously reported CATIE study, which emphasized the evaluation of treatment discontinuation rate [2], the present study focuses on the analysis of the treatment discontinuation rate, accompanied by analyses of secondary measures including remission rate and social activity, to evaluate the effectiveness of the study drugs. Considering that this study is randomized, interventional design, we set the study period as 104 weeks, enabling us to evaluate remission rate and improvement in social recovery in addition to the treatment discontinuation rate. The participants enrolled in this study also include the patients who received multiple antipsychotic medications. Switching to monotherapy is desirable from the viewpoints of improvement of adherence to treatment and alleviation of adverse reactions [26]. Thus, the present study plans to examine the possibility of switching from the preceding multiple antipsychotic medications to a single antipsychotic. In addition, the comprehensive capacity of individual patients is evaluated using the Japanese version of the MacCAT-CR, thus allowing the assessment of the influence of decision-making capacity on the continuation of treatment and improvement in social activity.

The evaluation of effectiveness afforded by this study is expected to yield new findings regarding the 3 study drugs for SGA selection for patients with schizophrenia.

We planned to conduct a randomized effectiveness study on aripiprazole, blonanserin, and paliperidone in patients with schizophrenia who had not received antipsychotic medication, in whom previous medication had been suspended, or who needed a switch from the current medication. The study period is 104 weeks. The primary endpoint is treatment discontinuation rate. Secondary endpoints are remission rate, improvement in social activity, adverse events. This study is expected to yield evidence for the selection of SGAs from the viewpoint of long-term outcome in patients with schizophrenia.

## Abbreviations

CGI-I: Clinical global impression-Improvement; CGI-SS: Clinical global Impression-suicide scale; DIEPSS: Drug-induced extrapyramidal symptoms scale; DSM-IV-TR: Diagnostic and statistical manual of mental disorders 4th edition text revision; EQ-5D: Euro quality of life-5 dimension; FGA: first-generation antipsychotics; GCP: Good clinical practice; MacCAT-CR: MacArthur competence assessment tool for clinical research; PANSS: Positive and negative syndrome scale; PSP: Personal and social performance scale; SGA: Second-generation antipsychotics.

## Competing interests

JI, TO and NI received research funding, grant, honoraria from Otsuka Pharmaceutical Co., Ltd., Dainippon Sumitomo Pharma Co., Ltd. and Janssen Pharmaceutical KK. KN received honoraria from Otsuka Pharmaceutical Co., Ltd., Dainippon Sumitomo Pharma Co., Ltd and Janssen Pharmaceutical KK. All authors have advisory role for Otsuka Pharmaceutical Co., Ltd., Dainippon Sumitomo Pharma Co., Ltd and Janssen Pharmaceutical KK.

## Authors’ contributions

All authors conceived the study and its design, and made the draft manuscript. All of them read and approved the final manuscript.

## Pre-publication history

The pre-publication history for this paper can be accessed here:

http://www.biomedcentral.com/1471-244X/13/243/prepub

## Data and safety monitoring committee

The Data and Safety Monitoring Committee consists of the following members: Teruhiko Higuchi, National Center of Neurology and Psychiatry, Japan; Mitsukuni Murasaki, Institute of CNS Psychopharmacology, Japan; and Kunitoshi Kamijima, International University of Health and Welfare, Japan.
